# Its Relation to Applicants for Training
*Read at Session I. of the Conference of the College of Nursing, held in the rooms of the Medical Society of London on Thursday, June 6.


**Published:** 1918-06-15

**Authors:** R. H. Gullan

**Affiliations:** Sister-Tutor, St. Thomas's Hospital.


### THE HIGHER EDUCATION OF NURSES.

### ITS RELATION TO APPLICANTS FOR TRAINING.*

By MISS R. H. GULLAN, Sister-Tutor, St. Thomas's Hospital.

The war has brought us up against many things, and not the least of these are the defects in our system of nursing that can produce such inequality of training and permit the curiously varying standard of efficiency found among the trained members of the profession. We are a heterogeneous body, with our general and special voluntary hospitals, large and small, our Poor-Law institutions, our District Nurses' Associations, our Private Nurses' Co-operations, our Nursing Homes; and we exist in self-contained and self-sufficing groups, holding aloof one from the other, so that we limit our usefulness and hamper our powers of expansion. As a body, lack of co-ordination is our weakness. Progress in one branch is nullified by retrogression in another, and efforts at reconstruction meet with almost insurmountable difficulties. For true progress in the nursing body all elements must be incorporated and harmoniously carried along to a higher level. Existing conditions outside our immediate sphere of influence must be recognised, as they cannot be swept away till a higher standard of living and education brings its own amelioration. For this reason reconstruction cannot closely follow the lines laid down in America, or even Australia, with their large standardised hospitals and absence of the diverse elements, but it can gain much by adopting their liberal outlook on the theoretical education of nurses.

### The Sense of Vocation.

The link that still binds all these diverse elements, as it has been the cause and motive power of their existence, is the sense of vocation that is deeply rooted in the hearts of British women, whatever their class or creed, and the higher education of nurses must mean rather the developing of that sense to its highest powers of understanding sympathy, and practical expression, by intellectual reasoning and a trained observation, than the accumulation of scientific knowledge as such. A higher education that rules out the desire for service, and offers only a highly scientific profession, where the instruction of the nurse comes before the well-being of the patient, is defeating its own ends. The sense of vocation is not always a conscious possession, especially in these days. It may be effectively replaced by something certainly less vivid and less malleable—a sense of duty and an earnestness of purpose—or it may even manifest only as a desire to do something useful. But with the careful fostering of ideals and the maintaining of a living interest, both may be developed to a very real enthusiasm and selfless devotion. And here the matron's influence is paramount. The portals of the nursing profession should be open to all who are urged thereto by an earnest desire to serve the sick and suffering, but a minimum standard of education must be set higher than has obtained at present, and it must be reached by those anxious to submit to the full training and gain the certificate of qualification.

### Approximation to a Uniform Standard.

While we have such varying demands to supply, and until life on all sides has reached a higher level to enable us to simplify the organisation of the nursing body, there must be training-schools adapted to the varying capacity of the applicants and that will provide instruction suitable to their best development, that the results may approximate as closely as possible to a uniform standard of efficiency. Thus candidates recruited from the so-called leisured and professional classes with high-school and college education or good private tuition, while being able more readily to accept theoretical instruction, must have the practical and economic side more carefully developed; while those who have had home cares and responsibilities enforcing a practical outlook on life, are usually endowed with much common sense, and adapt themselves readily to ward work, but are often sadly deficient in the education that will develop these powers to the highest usefulness and enable these girls to take positions of authority or to assume posts of administration. It is advisable that some schools should provide material that will attract the university student, and offer a training that will receive the support of headmistresses or college wardens, as providing a life-work worthy of the more developed mental powers of those under their guidance The profession of nursing has simply not existed in their thoughts as a possible career for their students. Yet, surely, our profession is worthy of the best brains and hearts. Granted these differences, it is universally agreed that a higher standard of theoretical knowledge should co-exist with the practical training accorded to all who enter the nursing institutions of Great Britain.

### An Invaluable Asset.

In all cases a preliminary training-school would be an invaluable asset to each large training-school, or a group of small institutions. It thoroughly tests the type of candidate, discovering her suitability for the work, and as to character and physical and mental powers. She is at once instructed along the desired lines as regards the ethics and practice of nursing. She would be taught the routine methods of ward work with scientific thoroughness, combining speed with efficiency; gain proficiency in invalid cooking; and obtain an elementary knowledge of anatomy, physiology, and hygiene, embracing also some simple domestic chemistry and physics. She can secure the knowledge without harassing stress of ward work, or it being bought by painful experience, often at the expense of her patient; and a good groundwork is laid for the future superstructure. Such preparation must greatly relieve the strain on the ward sister, and enable more time to be given to teaching details of nursing and imparting clinical knowledge.

### Facility without Understanding.

The first year is the most important in a nurse's training, for in it she lays her sound and sure foundation for a successful career. This first year should be chiefly given to supplementing the practice and experience of the wards with fuller explanatory detail, and description of other methods or their modification to suit altered conditions, laying stress on the principles involved to allow of greater flexibility and scope for creative thought. Hitherto nurses have been largely trained to accept methods and practices without question or without an understanding of their living meaning, and by force of constant repetition have produced mechanical imitations that in the hands of accurate observers become almost abuses, and

* Read at Session I. of the Conference of the College of Nursing, held in the rooms of the Medical Society of London on Thursday, June 6.

**The Higher Education of Nurses—(*continued*).**

gradually bad methods creep into the best training-schools. Each nurse, though taught to accept uniformity of practice for harmonious working in the ward, should be trained to reason out the methods that, under different conditions, she may create her own methods on sound lines. In this way growth is assured and progress made. The theory of practical nursing should be kept closely in touch with ward work. Actual cases and their treatment might be briefly discussed in class on the nurse's own report, while she can thus be guided as to accuracy of observation and clear, concise expression. The psychology of the patient should be brought to reckon constantly with the beneficial and healing force that lies in the right use of the powers of suggestion and the potent influence of a wholesome and harmonious atmosphere for the quick mental and physical recovery of her patient. But nothing can take the place of the knowledge gained by the actual experience in the ward.

### The Real Things of the Ward Nursing.

When all is said and done, such instruction can only supplement the ward teaching; it cannot replace it. It cannot give to the nurse the skilled art gained by practice under close supervision, nor those thousand-and-one details, independent of ordered treatment, that minister to the patient's well-being and happiness, that grow out of example rather than precept, that form the intangible "something" that distinguishes our British nurses all the world over. Those thousand-and-one details are the real things of the ward nursing: unseen by the doctors and, in most cases, unguessed at by them, not always possessed by the quick and smart nurse on her rounds, ready at response, alert at their special service, and who conveys the personal note that memory carries away and registers as "a good nurse," but they are the possession of her unobtrusive colleague impersonally serving the doctor's needs, and supplying the fine bond that brings the patient into the best relationship with the doctor. No text-book can convey such knowledge, which we, as British nurses, consider indispensable to a "good nurse," and which we pride ourselves is to be found in our wards. Hence, I believe, the comparative absence of nursing text-books from our literature.

### To Encourage Individual Thought.

In the first, the probationer's, year the theory and practice should cover the ground of general nursing that will provide suggestive material for full three years of training, and yet be well within the scope of understanding of a first-year nurse, for as her capacity expands with the experience gained in the wards and the knowledge accumulated from subsequent lectures by members of the medical and surgical staff, she will read more deeply into the meaning and will herself continually build on it. It is advisable to follow a method that will encourage individual thought, increase facility of accurate and concise expression, and one that will train to ordered thought and methodical arrangement, and the nurse should be required weekly to transcribe her own notes in a readable account and give a summary in her own words of the lectures she has attended. Such a method unconsciously trains for subsequent examination work by a very gradual process, while careful and full correction of these accounts should leave in the nurse's hands a useful working text-book, and one in which she must feel a real living interest. Many enter the profession whose powers of composition are very inadequate to the best use of the advantages a higher education offers, and as sound progress depends on the higher standard of the average candidate this difficulty must be dealt with at the outset, and it necessitates much heavy spade-work. Needless to say, time should be allowed from ward work for the class preparation.

### How Good Training Works.

The candidate who comes prepared by private tuition might often at the outset show to disadvantage in class work if compared with the candidate of secondary or even elementary schools, who would probably be better prepared for competitive work. But she has a more flexible mind and is easily instructed in the necessary details. Especially does she gain a remarkable facility if her mind has been enriched by social culture. Those who have benefited less by education find it uphill work, but conscientious application and the right spirit maintained over many months bring their reward, and they go forward to the competitive work of the second and third years almost as well equipped as the more efficiently educated ones. But if the spirit be lacking, the University graduate herself can make but a very sorry show in the final competitive tests, if these tests aim at a fine-blend of theory with practice. The first-year course must necessarily cover many months—in fact, the best part of the year—but the unrelaxed effort over such a long period trains the nurse in self-discipline, diligence, concentration, discrimination, reasoned thought, and deepens her interest and sense of responsibility. But failure attends this scheme unless the nurse be trained to think for herself, to give out to others what she gets, and can be brought to realise that each is responsible for the whole. The average mind is naturally lazy, and prefers to be spoon-fed, hence the individual note must always be fostered, but in due respect to community interests which, with advancing knowledge, should expand to the more corporate interests of the nursing world at large. During this period the unfolding of the nurse's mind, the development of her character and the habits she is forming in her ward work, are all portrayed in the material she submits weekly for correction, and through it she can be helped over difficulties, warned as to defects, and advised as to lines of action that will best develop her powers. In the smaller hospitals this post of teacher can most admirably be filled by the home sister, who has the very living interest of being daily in close contact with her pupils, and much of her guidance will be through the performance of her own duties when she exercises the double influence of example and precept. In the larger hospitals it would seem necessary to put this instruction in the hands of a special sister, fully trained, and if possible experienced in all methods of nursing, that the widest scope may be given to the nurse after her training to adapt herself to new conditions and new demands.

### The Second and Third Years.

In the second year the nurse will come under the instruction of members of the staff, receiving courses of lectures in anatomy, physiology, possibly chemistry and gynæcology. Free access to specimens from museum, dissecting-room, and laboratory would greatly increase the interest and practical knowledge of the nurse at these lectures, and it is the usual practice for the lecturers to avail themselves of these opportunities in a medical school. Revisional classes, supervision of note-books, and opportunities for clearing up difficult points should be provided by the sister in charge of the theoretical training. Each course should close with a written and *viva-voce* examination conducted by the lecturer himself.

**The Higher Education of Nurses—(*continued*).**

In the third year lectures will be given by the senior staff on surgical and medical nursing, followed by the usual examinations, and the nurse will then receive her final and exhaustive test in the theory and practice of general nursing, every opportunity being given during the *viva-voce* examination for a practical demonstration of efficiency, the necessary furniture, apparatus and appliances, etc., being at hand.

Having successfully passed this final test, the nurse may be said to be on the threshold of real effective work in the future, and stand firmer in the confidence of having satisfied the requirements of her training-school. Smaller hospitals can obtain much the same advantages by combining with others to procure the necessary courses of lectures and stimulate the competitive spirit. The progressive study thus followed should be continued in post-graduate lectures, which might deal with special branches, as diseases of children, orthopædic and ophthalmic nursing, skin diseases, electricity, and light treatments.

Post-Graduate Study.

From time to time during her training the nurse's interest might be aroused in the various activities of allied branches of her profession through lectures and discussions by those best fitted to deal with the subjects, such as district nursing, infant welfare, baby clinics, mothers' schools, industrial welfare, etc. Her life is so filled with the needs of the sick and suffering, and her thoughts and sympathies so continually engaged on the cure or alleviation of disease, that she is apt to forget the good old maxim "Prevention is better than cure," and may fail to appreciate the importance of those branches of preventive work: nay, she may even be intolerant of their outlook, when she should realise that workers in those branches are noble colleagues with whom she should walk hand-in-hand. Such subjects could be presented in greater detail at post-graduate classes, which would somewhat prepare the nurse herself for undertaking work if her inclination tended that way.

Finally, for those desirous of taking up teaching and administrative posts and the higher branches of the profession, a year's college course on household and social science, adapted to the requirements of the nursing profession, would greatly lessen the arduous task and shorten the period of toil at present necessitated if proficiency is to be gained by practical experience alone, unaided by scientific knowledge on the subjects.

The curriculum might advisably include such subjects as physiology, bacteriology, ethics, psychology, hygiene, economics, household work (including practical catering and nutrition), laundry, and bookkeeping, and a diploma might be granted to the successful student that would be recognised by the nursing world. Should entrance scholarships be attached to this course, much incentive would be given to the higher education of nurses, and lack of the necessary means would not debar those who could prove their capacity to benefit by such a course.

More Opportunity for Mental Culture.

A nurse's mind tends to become very narrowed in its interests and in its outlook on social and economic life. The three years of intensive culture necessary to her adequate training are so fully occupied with the demands of her hospital and her art—and her hours are still, unfortunately, so long—that her energies and sympathies are often exhausted. Also, hospital life, run, as it is, on lines wholly foreign to private life, is so utterly incomprehensible to the lay world that a nurse is, perforce, thrown back on her own *milieu* for understanding and sympathy, and the barrier is thus increased. For this reason sufficient consecutive time should be allotted from the hospital duties for her mental development and culture, and encouragement should be given to talent for sport within reasonable limits.

A Word to Private Nurses.

Private nurses would not run the gauntlet of so much criticism if they came prepared with a wider knowledge of their subject. Many enter the ranks still unformed in judgment outside the actual performance of their duties, and tact can only be acquired by knowledge of, and sympathy with, human nature. Private nursing is still, unfortunately, the best-paid branch of the profession, and the one procuring the greatest amount of variety, and it is thus apt to attract many in whom the nursing sense may not be strongly developed. It is difficult to retain ideals in private nursing, they get so little nourishment. All honour to those who do. I venture to suggest that much trouble would be avoided if it were made a practice for a nurse not to take up private nursing until she had filled some post of responsibility very suitably in a small hospital, where she would gain experience in administration and in the management of patients and their friends, and until she has shed the somewhat selfish garb worn throughout her training by assuming the full care of patients and of the training of probationers. District nurses have richly preserved the highest ideals of nursing, and are the sweet leavening of our body; but even they would benefit largely by a wider education, especially in preventive work, that might extend their beneficent influences over a wider field. An increased status automatically brings increased remuneration, as crying a necessity as the higher education of nurses, and one of the great causes of the retrogression of later years since nursing has become a profession as well as a vocation.

